# Neuritin promotes neurite and spine growth in rat cerebellar granule cells via L‐type calcium channel‐mediated calcium influx

**DOI:** 10.1111/jnc.14535

**Published:** 2018-08-16

**Authors:** Qian‐Ru Zhao, Jun‐Mei Lu, Zhao‐Yang Li, Yan‐Ai Mei

**Affiliations:** ^1^ Institutes of Brain Science State Key Laboratory of Medical Neurobiology and School of Life Sciences Fudan University Shanghai China

**Keywords:** Ca^2+^‐CaN‐NFATc4 axis, cerebellar granule neurons, insulin receptor, L‐type calcium channel, neurite and spine growth, neuritin

## Abstract

Neuritin is a neurotrophic factor that is activated by neural activity and neurotrophins. Its major function is to promote neurite growth and branching; however, the underlying mechanisms are not fully understood. To address this issue, this study investigated the effects of neuritin on neurite and spine growth and intracellular Ca^2+^ concentration in rat cerebellar granule neurons (CGNs). Incubation of CGNs for 24 h with neuritin increased neurite length and spine density; this effect was mimicked by insulin and abolished by inhibiting insulin receptor (IR) or mitogen‐activated protein kinase kinase/extracellular signal‐regulated kinase (ERK) activity. Calcium imaging and western blot analysis revealed that neuritin enhanced the increase in intracellular Ca^2+^ level induced by high K^+^, and stimulated the cell surface expression of Ca_V_1.2 and Ca_V_1.3 α subunits of the L‐type calcium channel, which was suppressed by inhibition of IR or mitogen‐activated protein kinase kinase/ERK. Treatment with inhibitors of L‐type calcium channels, calmodulin, and calcineurin (CaN) abrogated the effects of neuritin on neurite length and spine density. A similar result was obtained by silencing nuclear factor of activated T cells c4, which is known to be activated by neuritin in CGNs. These results indicate that IR and ERK signaling as well as the Ca^2+^/CaN/nuclear factor of activated T cells c4 axis mediate the effects of neuritin on neurite and spine growth in CGNs.

**Open Practices:**

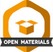

Open Science: This manuscript was awarded with the Open Materials Badge.

For more information see: https://cos.io/our-services/open-science-badges/

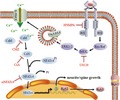

Cover Image for this issue: doi: 10.1111/jnc.14195.

**Abbreviations used**
[Ca^2+^]_i_: intracellular calcium (Ca^2+^) concentration
CaM: calmodulin
CaN: calcineurin
CGNs: cerebellar granule neurons
CMZ: calmidazolium chloride
CsA: cyclosporin A
ERK: extracellular signal‐regulated kinase
Fura‐2 AM: Fura‐2 acetoxymethyl ester
HK: high potassium (K^+^)
HNMPA: hydroxy‐2‐naphthalenylmethyl phosphonic acid
*I*_A_: transient K^+^ outward current
*I*_Ca_: calcium current
IR: insulin receptor
MEK: mitogen‐activated protein kinase kinase
NFAT: nuclear factor of activated T cells
VGCC: voltage‐gated calcium channel

Neuritin is a neurotrophic factor activated by neural activity and neurotrophins (Nedivi *et al*. 1993; Naeve *et al*. 1997). Neuritin has multiple roles in neural development, including synaptic plasticity and maturation and neurite outgrowth (Naeve *et al*. 1997; Son *et al*. 2012). Neuritin is also involved in neuronal apoptosis and regeneration after ischemia and traumatic brain injury (Rickhag *et al*. 2007; He *et al*. 2013; Yan *et al*. 2016). Although it is known to exert non‐cell‐autonomous effects by binding to receptors (Nedivi *et al*. 1998; Cantallops *et al*. 2000), a specific neuritin receptor has yet to be identified, and the downstream effectors are unknown. We previously reported that neuritin stimulated the expression of transient potassium (K^+^) outward current (*I*_A_) channel K_V_4.2 subunits in rat cerebellar granule neurons (CGNs) via insulin receptor (IR) activation and mitogen‐activated protein kinase kinase (MEK)/extracellular signal‐regulated kinase (ERK) and Akt/mammalian target of rapamycin signaling pathways (Yao *et al*. 2012), and that activation of the Ca^2+^/CaN/nuclear factor of activated T cells (NFAT)c4 axis was required for this effect (Yao *et al*. 2016). We also showed that activation of IR and MEK/ERK signaling by neuritin enhanced synaptic transmission in the medial prefrontal cortex (Lu *et al*. 2017).

Neuritin was first described as a factor that promotes neuritogenesis (Naeve *et al*. 1997). Treatment of primary rat hippocampal and cortical neuron cultures with recombinant neuritin for 4 days induced extensive neuritogenesis (Naeve *et al*. 1997). Similar effects have been reported in PC12 cells and motor neurons (Marron *et al*. 2005; Cappelletti *et al*. 2007) as well as in hippocampal neurons in depression models, in which neuritin over‐expression in the hippocampus prevented the atrophy of dendrites and dendritic spines under conditions of chronic stress (Son *et al*. 2012). We recently showed that neuritin over‐expression increased dendritic spine density in mouse hippocampal neurons and reversed deficits in novel object associative recognition memory in mice caused by exposure to extremely low frequency (50 Hz) electromagnetic fields (Zhao *et al*. 2015). However, the mechanistic basis for the effects of neuritin has yet to be established.

CGNs are the most abundant neurons in the brain (Herculano‐Houzel 2010). Cultured CGNs typically have a round soma and extensive network of neurites (Powell *et al*. 1997), and have been widely used to study the mechanisms of neuronal survival, programmed cell death (Borodinsky *et al*. 2002; Zhou *et al*. 2012), and neurite and synapse development (Borodinsky *et al*. 2002; Sato *et al*. 2005). The latter was shown to be modulated by high K^+^ (HK) depolarization‐induced Ca^2+^ signaling (Borodinsky *et al*. 2002; Sato *et al*. 2005), while voltage‐gated calcium channel (VGCC) activation induced neurite growth, which was mediated by Ca^2+^/calmodulin (CaM)‐dependent protein kinase II in immature CGNs (Borodinsky *et al*. 2002; Gaudilliere *et al*. 2004). We also observed that neuritin increased intracellular Ca^2+^ concentration ([Ca^2+^]_i_) and HK‐induced Ca^2+^ influx in mouse CGNs and activated the Ca^2+^/CaN/NFATc4 axis, leading to up‐regulation of K_V_4.2 protein (Yao *et al*. 2016). Whether neuritin promotes neurite and spine growth in CGNs by increasing [Ca^2+^]_i_ and whether the CaN/NFATc4 axis is involved remain to be determined.

In this study, we used rat CGNs to evaluate the effects of neuritin on neurite and spine growth and [Ca^2+^]_i_. We also examined whether IR and ERK signaling pathways and the Ca^2+^/CaN/NFATc4 axis mediate the effects of neuritin.

## Materials and methods

### Chemicals

Recombinant human neuritin (cat. no. CH42) was purchased from Novoprotein (Shanghai, China). Poly‐l‐lysine (cat. no. P2636), cytosine 1‐beta‐d‐arabinofuranoside (cat. no. P6645), tetraethylammonium chloride (cat. no. T2265), tetrodotoxin (cat. no. A803467), 4‐aminopyridine (4‐AP) (cat. no. A78403), insulin (cat. no. I‐4011), calmidazolium chloride (CMZ) (cat. no. C3930), and cyclosporin A (CsA) (cat. no. C‐139) were from Sigma‐Aldrich (St. Louis, MO, USA, RRID:SCR_008988). Dulbecco's modified Eagle's medium (cat. no. 12100‐046), fetal bovine serum (cat. no. 16000‐044), and antibiotic‐antimycotic solution (cat. no. 15240062) were from Gibco (Rockville, MD, USA, RRID:SCR_008817). Hydroxy‐2‐naphthalenylmethyl phosphonic acid (HNMPA) (cat. no. sc‐205714) was from Santa Cruz Biotechnology (Dallas, TX, USA, RRID:SCR_008987), and U0126 monoethanolate (U0126) (cat. no. S1102) was from Selleck Chemicals (Houston, TX, USA, RRID:SCR_003823).

### Cell culture

The procedure was approved by the Committee on the Ethics of Animal Experimentation at Fudan University (Permit No. 20090614‐001). CGNs were obtained from the cerebella of 7‐day‐old Sprague–Dawley female rat pups (average weight 180 g, SLAC Laboratory Animal Co., Shanghai, China) as following steps. Briefly, the meninges and vessels of the cerebellum were removed in ice‐cold Kreb‐Ringer solution (120 mM NaCl, 25 mM NaHCO_3_, 14.3 mM glucose, 4.8 mM KCl, 2.5 mM MgSO_4_, 1.2 mM KH_2_PO_4_, and 0.3% w/v bovine serum albumin) after decapitation. The tissues were minced with scissors, resuspended in the same solution and centrifuged at 2000 rpm for 1 min. The supernatant was discarded and the pellet was resuspended in Kreb‐Ringer solution containing 0.025% w/v trypsin (cat. no. T8003; Sigma‐Aldrich) and digested at 25 °C for 6 min. The digestion was terminated by adding 0.1% w/v trypsin inhibitor (cat. no. T9128; Sigma‐Aldrich), 0.008% w/v DNAse I (cat. no. 10104159001; Roche, Milano, Italy, RRID:SCR_008987), and 3.2 mM MgSO_4_ followed by centrifugation (900 *g* for 1 min). The sediment was triturated until there were no visible clusters of tissue. Kreb‐Ringer solution containing 2.5 mM MgSO_4_ and 0.1 mM CaCl_2_ was then added to the cell suspension, followed by centrifugation at 400 *g* for 3 min. The sediment was resuspended in fresh culture medium consisting of Dulbecco's modified Eagle's medium supplemented with 10% fetal bovine serum, 25 mM KCl, and 1% antibiotic‐antimycotic solution and seeded in 35‐mm Petri dishes (cat. no. 010035; Shenyou, Shanghai, China) coated with poly‐l‐lysine (10 g/mL) or on coverslips (cat. no. 12‐545‐100; Fisher brand, Leicestershire, UK) coated with poly‐l‐lysine (50 g/mL) at a density of 10^5^ cells/cm^2^. Cells were cultured on coverslips dotted with paraffin at a low density (50–130 cells/mm^2^) for morphological analysis (Kubota *et al*. 2013). At 6 h after plating, the coverslips were turned over onto cells cultured at a normal density (Kubota *et al*. 2013) followed by incubation at 37°C and 5% CO_2_ in the culture medium. Cytosine 1‐beta‐d‐arabinofuranoside (5 μM) was added to the culture medium 24 h after seeding to inhibit the proliferation of non‐neuronal cells. Cells were used for experiments after culturing for 3–5 days unless otherwise indicated.

### Cell membrane staining

Cells were stained with 1,1’‐dioctadecyl‐3,3,3’,3’‐tetramethylindodicarbocyanine, 4‐chlorobenzenesulfonate salt (DiD) as previously described (Wei *et al*. 2011). Briefly, cells grown on coverslips were fixed with 4% paraformaldehyde in phosphate‐buffered saline (PBS) at 25 °C. After washing with PBS, the cells were stained with Vybrant DiD cell‐labeling solution (1 : 200; cat. no. V22887; Invitrogen, Carlsbad, CA, USA, RRID:SCR_008410) at 37°C. The cells were washed with warm PBS and incubated in PBS at 4°C for 24 to 48 h to allow incorporation of DiD into the cell membrane.

### Analysis of neuronal morphology

The neuronal morphology was analyzed with double blind method. Samples were arbitrarily allocated. The investigator who captured the pictures on a confocal microscope (LSM700; Zeiss, Oberkochen, Germany) had no information about sample treatments. The images were analyzed by another investigator who was also blinded to the group assignment. A homogenous population of neurons was selected for neurite and spine measurements based on the following criteria: (i) the cell body and neurites were completely impregnated with dye; (ii) selected neurons were separate from surrounding neurons; and (iii) all of the neurites were visible within the plane of focus. Neurite lengths and branches of selected cells were reconstructed using NIH ImageJ software (RRID:SCR_003070) (Cho *et al*. 2017). Sholl analysis was carried out to quantify the number of branch intersections (Yao *et al*. 2016). Spine density is expressed as the average number of spines per 100 μm of neurite. Spines with a stubby or mushroom shape were regarded as mature, while those with a thin morphology were considered immature (Wei *et al*. 2011). A mushroom spine was defined as a neurite protrusion with a head size 50% larger than the neck (Wei *et al*. 2011).

### Measurement of [Ca^2+^]_i_ levels in CGNs

CGNs grown on coverslips were loaded with Fura‐2 acetoxymethyl (AM) ester (cat. no. F1201; Invitrogen) and 0.02% pluronic F127 (cat. no. P3000MP; Invitrogen) in Hanks’ balanced salt solution (HBSS) composed of 126.0 mM NaCl, 2.5 mM KCl, 2.0 mM MgSO_4_, 2.0 mM CaCl_2_, 10.0 mM d‐glucose, and 10.0 mM HEPES (pH 7.4) at 37°C for 45 min in the dark (Yao *et al*. 2016). The cells were rinsed three times in Fura‐2 AM‐free HBSS at 25 °C. The coverslips were transferred to an open slide chamber containing 1 mL of Fura‐2 AM‐free HBSS that was placed on an inverted epifluorescence microscope (Eclipse Ti; Nikon, Tokyo, Japan). Excitation and emission wavelengths for Fura‐2 AM were 340/380 and 505 nm, respectively. Baseline [Ca^2+^]_i_ was determined for 60 s prior to adding the HK solution (40 mM KCl) (Chen *et al*. 2005). Data were acquired at 4‐s intervals throughout the experiment. Fluorescence intensity was quantified using MetaFluor software (RRID:SCR_014294). Calibration for calcium imaging was performed *in vitro* using a calcium calibration buffer kit (cat. no. F6774; Invitrogen) (Velazquez‐Marrero *et al*. 2014). The results were plotted according to the equation [Ca^2+^]_free_ = *K*_d_ × [*R* − *R*_min_]/[*R*_max_ − *R*] × F380 max/F380 min, where *R* is the ratio of 505 nm emission intensity under 380 nm excitation to 505 nm emission under 340 nm excitation; *R*_min_ is the same ratio at [Ca^2+^]_free_ = 0; *R*_max_ is the ratio at saturating [Ca^2+^]; F380 max refers to the fluorescence intensity with excitation at 380 nm for [Ca^2+^]_free_ = 0; and F380 min refers to the fluorescence intensity at saturating [Ca^2+^]_free_. After linear fitting, *K*_d_ was determined from the standard line and [Ca^2+^] corresponding to *R* was calculated using the equation.

### Patch clamp recording

Whole‐cell currents of CGNs were recorded at 25 °C using a standard patch‐clamping technique (Lu *et al*. 2016). Prior to calcium current (*I*_Ca_) recording, the culture medium was replaced with a solution composed of 147 mM tetraethylammonium chloride, 10 mM BaCl_2_, 10 mM HEPES (pH 7.4), 2 mM MgCl_2_, 1 μM tetrodotoxin, 2 mM 4‐AP, and 10 mM glucose. Soft glass recording pipettes were filled with a solution composed of 145 mM CsCl, 10 mM EGTA, 10 mM HEPES (pH 7.3), 5 mM Na_2_‐ATP, and 0.5 mM Na_2_‐GTP. The resistance of the pipette filled with internal solution was 4–6 MΩ. Current responses were low‐pass filtered at 1 kHz. The P/4 protocol was used to subtract the linear leak and capacitative currents, which were initiated from a holding potential of −80 mV.

### Biotin labeling

Proteins on the cell surface were visualized by biotin labeling as previously described (Zhan *et al*. 2014). Cultured cells were washed with PBS, then incubated with 0.25 mg/mL EZ‐Link sulfo‐N‐hydroxysuccinimide‐SS‐biotin (cat. no. 21331; Thermo Fisher Scientific, Rockford, IL, USA, RRID:SCR_008452) in PBS for 40 min at 4°C. Sulfo‐N‐hydroxysuccinimide‐SS‐biotin solution was replaced with 10 mM glycine (pH 8.0) in PBS and incubated for 20 min at 4°C to block the reaction. After washing three times with ice‐cold PBS, cells were lysed with HEPES‐Nonidet (N)P‐40 lysis buffer composed of 150 mM NaCl, 50 mM NaF, 20 mM HEPES (pH 7.5), 2 mM EDTA, 100 μM Na_3_VO_4_, 10% glycerol, 0.5% NP‐40, and 1% protease inhibitor cocktail (cat. no. P8340; Sigma, St Louis, MO, USA) for 30 min. After centrifugation at 19 600 *g* for 10 min, the supernatant was mixed with streptavidin‐agarose beads (cat. no. 20347; Pierce, Rockford, IL, USA, RRID:SCR_013270) overnight at 4°C. The mixture was washed with HEPES‐NP‐40 lysis buffer and centrifuged at 4900 *g* four times; the pellet was mixed with 1× sodium dodecyl sulfate (SDS) loading buffer, boiled at 50°C for 20 min, and centrifuged, and the supernatant was analyzed.

### Western blotting

Cultured cells were lysed in HEPES‐NP‐40 lysis buffer on ice for 30 min. After centrifugation at 14 400 *g* for 15 min, the supernatant was mixed with 2× SDS loading buffer and boiled at 95°C for 5 min. Before loading, total protein concentration was determined using a microplate spectrophotometer (MultiSKAN MK3; Thermo Fisher Scientific). Equal amounts of protein were loaded in the 10% acrylamide gel and separated by SDS‐polyacrylamide gel electrophoresis, then transferred to a polyvinylidene difluoride membrane (Millipore, Billerica, MA, USA, RRID:SCR_008983) that was blocked with 10% non‐fat milk and incubated overnight at 4°C with the following antibodies: mouse monoclonal anti‐Ca_V_1.2 (RRID:AB_10673150) and ‐Ca_V_1.3 (RRID:AB_10672111) (both 1 : 500; University of California, Davis, CA, USA) (Lu *et al*. 2016), rabbit polyclonal anti‐NFATc4 (RRID:AB_650208) (1 : 1000; Santa Cruz Biotechnology) (Yao *et al*. 2016), rabbit polyclonal anti‐Na‐K ATPase (1 : 1000; cat. no. 14418‐1‐AP; Proteintech, Rosemont, IL, USA, RRID: SCR_008986), and mouse monoclonal anti‐glyceraldehyde 3‐phosphate dehydrogenase (GAPDH) (1 : 10 000; cat. no. KC‐5G4; Kang Chen Bio‐Tech, Shanghai, China). After washing three times for 45 min in Tris‐buffered saline with 0.3% Tween‐20, the membrane was incubated for 2 h at 25 °C with horseradish peroxidase‐conjugated anti‐mouse (cat. no. A0216) or ‐rabbit IgG (cat. no. A0208) (both 1 : 1000; Beyotime Institute of Biotechnology, Haimen, China). Protein bands were detected by enhanced chemiluminescence using a Super Signal West Pico trial kit (cat. no. 34080; Pierce) and were visualized with the ChemiDoc XRS system (Bio‐Rad, Hercules, CA, USA, RRID:SCR_013553). ImageLab software (Bio‐Rad) was used for background subtraction and protein quantification. The linearity of the western blots was confirmed in separate experiments.

### Lentiviral transfection

A lentivirus encoding short interfering RNA targeting NFATc4 (siNFATc4) with the sequence 564‐gggacggctctcctagagatt‐584 (Benedito *et al*. 2005; Vashishta *et al*. 2009) was used to knock down NFATc4 expression. The lentivirus expressing scramble sequence 5’‐ttctccgaacgtgtcacgt‐3’ was used as control. The lentivirus was generated by Genechem (Shanghai, China), with a titer of 10^9^/mL and multiplicity of infection of 100. CGNs were transfected with lentivirus for 3 days and then lysed for western blotting or stained by DiD solution.

### Data acquisition

Origin 8.0 (RRID:SCR_014212) was used to analyze the data. The size of the samples was determined by power analysis. The results of Kolmogorov–Smirnov test indicated that all samples except for the neurite branching result were normally distributed. The Student's *t*‐test was used to compare two samples and one‐way analysis of variance (anova) with Bonferroni's post hoc test was used for comparisons between multiple groups. Neurite branching was analyzed by Friedman test, with drug treatment and distance from the soma as two factors. Data out of the range of mean ± 3 SD were regarded as outliers and discarded. Data are presented as mean ± SEM, with *n* representing the number of neurons used for morphological analysis, calcium imaging, or electrophysiological recordings. Western blotting data are presented as individual data points in a scatter plot, with *n* representing the number of experiments. For electrophysiology, data were collected from at least four different batches of neurons prepared on different days to minimize bias resulting from culture conditions. *p* ≤ 0.05 was considered statistically significant in all statistical tests.

## Results

### Neuritin increases neurite length and spine density by activating IR and MEK/ERK signaling pathways in CGNs

**Figure 1:**
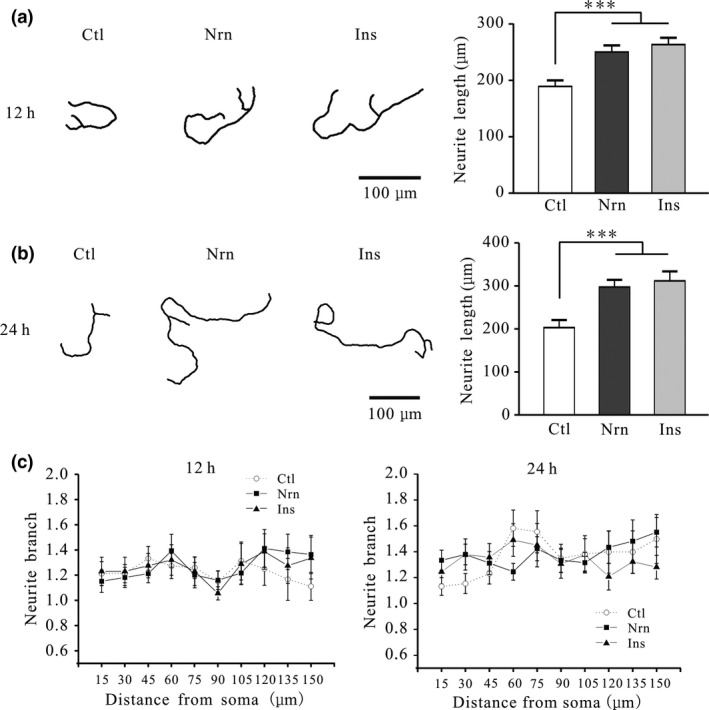
Neuritin and insulin increase neurite length in rat cerebellar granule neurons (CGNs) without affecting branching. (a) Effects of neuritin on neurite length in CGNs after treatment for 12 h. Left panel shows representative images of control (Ctl) cells, and cells treated with neuritin (Nrn) or insulin (Ins); right panel shows the quantitative analysis (Ctl, *n* = 43; Nrn, *n* = 48; Ins, *n* = 35). (b) Representative images and quantitative analysis of the effects of neuritin on CGN neurite length after treatment for 24 h (Ctl, *n* = 41; Nrn, *n* = 52; Ins, *n* = 33). (c) Quantitative analysis of branch intersections after neuritin treatment for 12 h (Ctl, *n* = 33; Nrn, *n* = 33; Ins, *n* = 22) or 24 h (Ctl, *n* = 45; Nrn, *n* = 45; Ins, *n* = 45) determined by Sholl analysis and Friedman test. Results represent mean ± SEM, *n* indicates the number of cells. ***p < 0.001 between groups connected with a straight line (one‐way anova followed by Bonferroni post hoc test).

Neuritin has been shown to promote the growth of neurites in hippocampal and cortical neurons (Naeve *et al*. 1997). We therefore investigated whether neuritin promotes the growth of neurites in CGNs by evaluating neurite length and number of branches. Incubation of CGNs with 150 ng/mL neuritin increased neurite length by 32.2 ± 6.1% after 12 h (*n* = 43 and 48, *p* < 0.001) and by 46.6 ± 8.4% after 24 h (*n* = 41 and 52, *p* < 0.001) (. 1a and b). Similar effects were obtained by treatment with 150 ng/mL insulin, with increases in neurite length of 39.3 ± 6.0% and 53.6 ± 9.7% observed after 12 and 24 h, respectively (*n* = 35 and 33, *p* < 0.001) (Fig. 1a and b). Sholl analysis and Friedman test revealed that neither neuritin nor insulin affected neurite branching after 12 or 24 h and the interactions between distance from the soma and the drug treatment were not significantly different (Fig. 1c).

**Figure 2:**
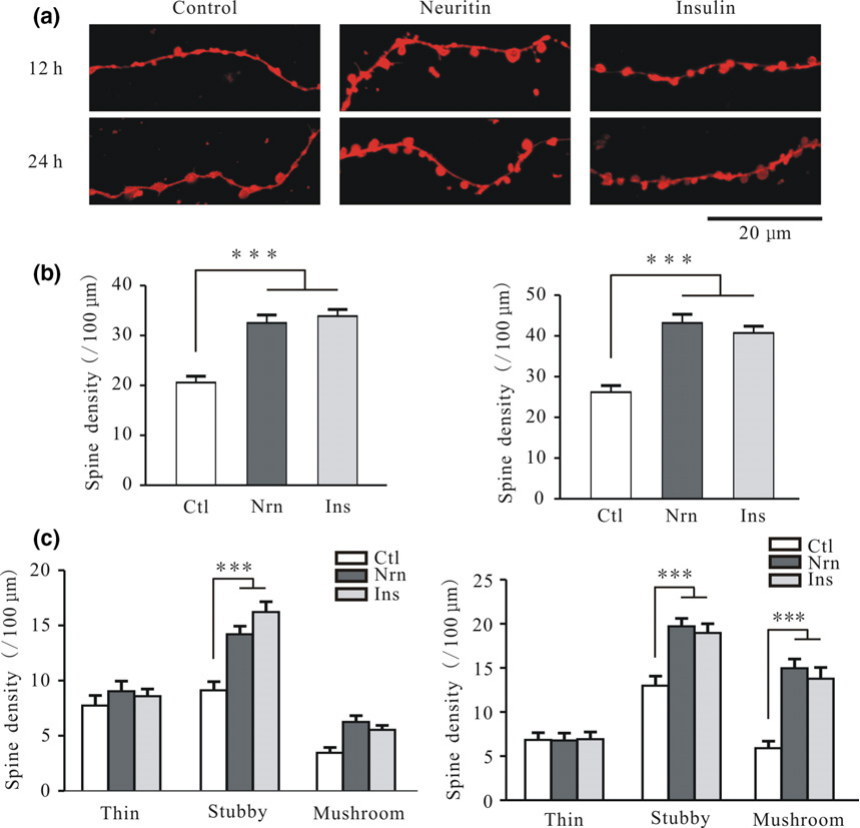
Neuritin and insulin increase neurite spine density in cerebellar granule neurons (CGNs). (a) Representative images of the effects of neuritin and insulin on CGN spine number after treatment for 12 or 24 h. (b) Statistical analysis of the effects of neuritin on CGN spine density after treatment for 12 h (left) (Ctl, *n* = 32; Nrn, *n* = 33; Ins, *n* = 32) or 24 h (right) (Ctl, *n* = 28; Nrn, *n* = 35; Ins, *n* = 25). (c) Statistical analysis of the effects of neuritin treatment for 12 h (left) (Ctl, *n* = 33; Nrn, *n* = 39; Ins, *n* = 33) or 24 h (right) (Ctl, *n* = 37; Nrn, *n* = 40; Ins, *n* = 41) on thin, stubby, and mushroom spines. Results represent mean ± SEM, *n* indicates the number of cells. ***p < 0.001 between groups connected with a straight line (one‐way anova followed by Bonferroni post hoc test).

In our previous study, we demonstrated that over‐expressing neuritin in the hippocampus and prefrontal cortex increased spine density in pyramidal neurons (Zhao *et al*. 2015; Yao *et al*. 2016). Here we found that incubation of CGNs with 150 ng/mL neuritin for 12 and 24 h increased spine density by 58.0 ± 7.0% (*n* = 32 and 33, *p* < 0.001) and 64.9 ± 7.3% (*n* = 28 and 35, *p* < 0.001), respectively (Fig. 2a and b). Similarly, treatment with 150 ng/mL insulin for 12 and 24 h increased total spine density by 64.7 ± 7.0% (*n* = 32, *p* < 0.001) and 55.4 ± 6.3% (*n* = 25, *p* < 0.001), respectively (Fig. 2a and b).

As in the case of hippocampal and cortical neurons, the spines of CGN neurites can be classified as thin, stubby, and mushroom‐shaped according to the ratio of spine head to spine neck width (Wei *et al*. 2011). We evaluated the effects of neuritin on the three types of spine and found that it had no effect on thin spines after 12 or 24 h (Fig. 2c). However, the density of stubby spines was increased by 77.8 ± 9.5 % (*n* = 33 and 39, *p* < 0.001) and 46.1 ± 8.2% (*n* = 37 and 40, *p* < 0.001) after 12 and 24 h, respectively (Fig. 2c). Although neuritin treatment did not significantly affect the density of mushroom spines after 12 h, an increase of 133.0 ± 18.1% was observed after 24 h (*n* = 36 and 39, *p* < 0.001) (Fig. 2c). Insulin treatment for 12 h increased the density of stubby spines by 55.6 ± 8.2% (*n* = 33 and 33, *p* < 0.001) (Fig. 2c); however, after 24 h, the density of stubby and mushroom spines was increased by 51.9 ± 7.7% (*n* = 37 and 41, *p* < 0.001) and 153.1 ± 15.5% (*n* = 36 and 40, *p* < 0.001), respectively (Fig. 2c).

**Figure 3:**
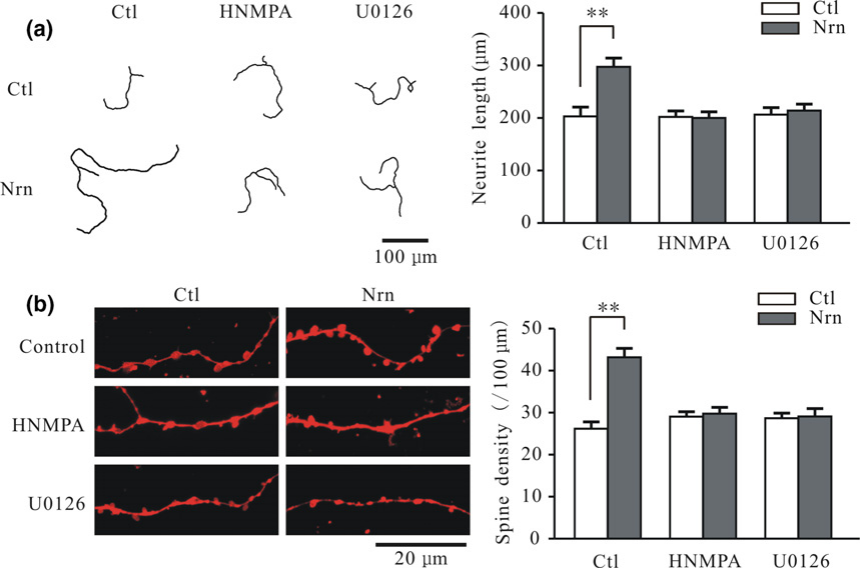
Blocking insulin receptor (IR) and extracellular signal‐regulated kinase (ERK) activation inhibits neuritin‐induced increases in cerebellar granule neuron (CGN) neurite length and spine density. (a) Representative images (left) and quantitative analysis (right) of the effects of neuritin on neurite length in CGNs cultured in the absence and presence of the IR blocker hydroxy‐2‐naphthalenylmethyl phosphonic acid (HNMPA) and ERK inhibitor U0126 (Ctl, *n* = 41; Nrn, *n* = 52; HNMPA, *n* = 41; HNMPA + Nrn, *n* = 43; U0126, *n* = 38; U0126 + Nrn, *n* = 43). (b) Representative images and quantitative analysis of the effects of neuritin on total spine density in CGNs cultured in the absence and presence of the IR blocker HNMPA and ERK inhibitor U0126 (Ctl, *n* = 28; Nrn, *n* = 35; HNMPA, *n* = 31; HNMPA + Nrn, *n* = 26; U0126, *n* = 22; U0126 + Nrn, *n* = 21). Results represent mean ± SEM, *n* indicates the number of cells. **p < 0.01 between groups connected with a straight line (one‐way anova followed by Bonferroni post hoc test).

We previously reported that neuritin‐activated IR and MEK/ERK signaling leads to up‐regulation of K_V_4.2 subunits in CGNs and enhanced synaptic transmission in the medial prefrontal cortex (Yao *et al*. 2012; Lu *et al*. 2017). To determine whether neuritin‐induced increases in neurite length and spine density in CGNs involve IR and MEK/ERK signaling pathways, we used HNMPA and U0126 to block IR and ERK activity, respectively. Treatment with 100 μM HNMPA (Yao *et al*. 2016), neuritin did not significantly increase neurite length (1.0 ± 5.7%, *n* = 41 and 43) or spine density (2.4 ± 4.6%, *n* = 31 and 26) relative to the control (*p* > 0.05) (Fig. 3a and b). On the other hand, 10 μM U0126 (Lu *et al*. 2017) abrogated the neuritin‐induced increases in neurite length and total spine density to 3.7 ± 6.2% (*n* = 38 and 43) and 1.5 ± 5.4% (*n* = 22 and 21) of the control values, respectively (Fig. 3a and b).

### Neuritin increases Ca^2+^ influx by inducing membrane expression of L‐type Ca^2+^ channel

**Figure 4:**
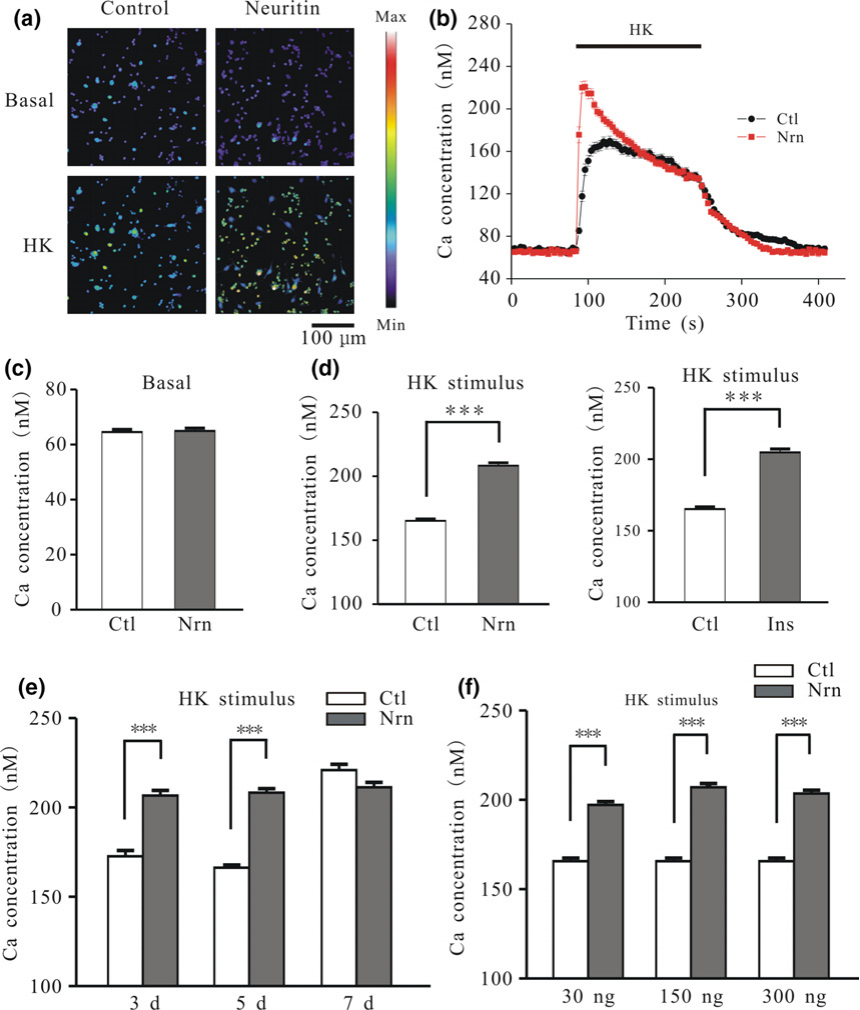
Neuritin and insulin increase Ca^2+^ influx induced by high potassium (HK) depolarization in a time‐dependent manner. (a) Effects of neuritin on [Ca^2+^]_i_ before (basal) and after high potassium (K^+^) (HK) depolarization. Changes in Fura‐2 AM fluorescence excitation ratios with increasing [Ca^2+^]_i_ are represented as a color change from purple to red. (b) Changes in [Ca^2+^]_i_ upon application of a depolarizing HK solution in the absence or presence of neuritin. (c) Quantitative analysis of the effects of neuritin on basal [Ca^2+^]_i_ (Ctl, *n* = 137; Nrn, *n* = 121). (d) Quantitative analysis of [Ca^2+^]_i_ induced by HK in the absence or presence of neuritin and insulin (Ctl, *n* = 120; Nrn, *n* = 120; Ins, *n* = 82). (e) Comparison of the effects of neuritin on [Ca^2+^]_i_ induced by HK after different lengths of time in culture (days) (3 days Ctl, *n* = 79; 3 days Nrn, *n* = 97; 5 days Ctl, *n* = 128; 5 days Nrn, *n* = 120; 7 days Ctl, *n* = 48; 7 days Nrn, *n* = 54). (f) Effects of neuritin concentration on [Ca^2+^]_i_ induced by HK (Ctl, *n* = 116; 30 ng/mL Nrn, *n* = 109; 150 ng/mL Nrn, *n* = 124; 300 ng/mL Nrn, *n* = 125). Data represent mean ± SEM, *n* indicates the number of cells. ***p < 0.001 between groups connected with a straight line (unpaired t test or one‐way anova followed by Bonferroni post hoc test).

Increased [Ca^2+^]_i_ is associated with neurite and synapse growth (Borodinsky *et al*. 2002; Gaudilliere *et al*. 2004; Sato *et al*. 2005). Here, we examined whether neuritin increases [Ca^2+^]_i_ by Ca^2+^ imaging using the fluorescent dye Fura‐2 AM. Neuritin did not affect basal [Ca^2+^]_i_ in rat CGNs treated with 150 ng/mL neuritin for 20 min (Fig. 4a–c), but increased Ca^2+^ influx induced by HK depolarization solution, which activated VGCCs and induced a rapid increase in [Ca^2+^]_i_ (Fig. 4a, b, and d). In control neurons, depolarization with HK caused an acute elevation of [Ca^2+^]_i_ from 64.5 ± 0.9 to 165.7 ± 1.4 nM (*n* = 120). Neuritin treatment increased the maximum [Ca^2+^]_i_ to 208.4 ± 2.2 nM (*n* = 120) (Fig. 4b and d). Insulin has similar effects, with the HK‐induced peak [Ca^2+^]_i_ increasing to 204.8 ± 2.4 nM (*n* = 82) relative to the control upon treatment with 150 ng/mL insulin (*p* < 0.001) (Fig. 4d).

We next examined whether the effects of neuritin on the HK‐induced increase in [Ca^2+^]_i_ was related to developmental stage or neuritin concentration. Treatment with 150 ng/mL neuritin increased the HK‐induced [Ca^2+^]_i_ concentration in CGNs after 3 days in culture (DIC) from 172.6 ± 3.2 to 206.7 ± 2.9 nM (*n* = 79 and 97, *p* < 0.001) and after 5 DIC from 166.4 ± 1.5 to 208.4 ± 2.2 nM (*n* = 128 and 120, *p* < 0.001), but not after 7 DIC (from 220.9 ± 3.3 to 211.4 ± 2.7 nM, *n* = 48 and 54, *p* > 0.05) (Fig. 4e). [Ca^2+^]_i_ after depolarization with HK was increased in the presence of neuritin at concentrations of 30 ng/mL (197.2 ± 1.9 nM, *n* = 109, *p* < 0.001), 150 ng/mL (207.0 ± 2.2 nM, *n* = 124, *p* < 0.001), and 300 ng/mL (203.6 ± 1.9 nM, *n* = 125, *p* < 0.001) relative to the control group (165.8 ± 1.8 nM, *n* = 116) (Fig. 4f). The difference between 30 and 150 ng/mL was statistically significant (*p* < 0.01), but there was no difference between the 150 and 300 ng/mL groups (*p* > 0.05), indicating that 150 ng/mL is a reasonable treatment concentration.

**Figure 5:**
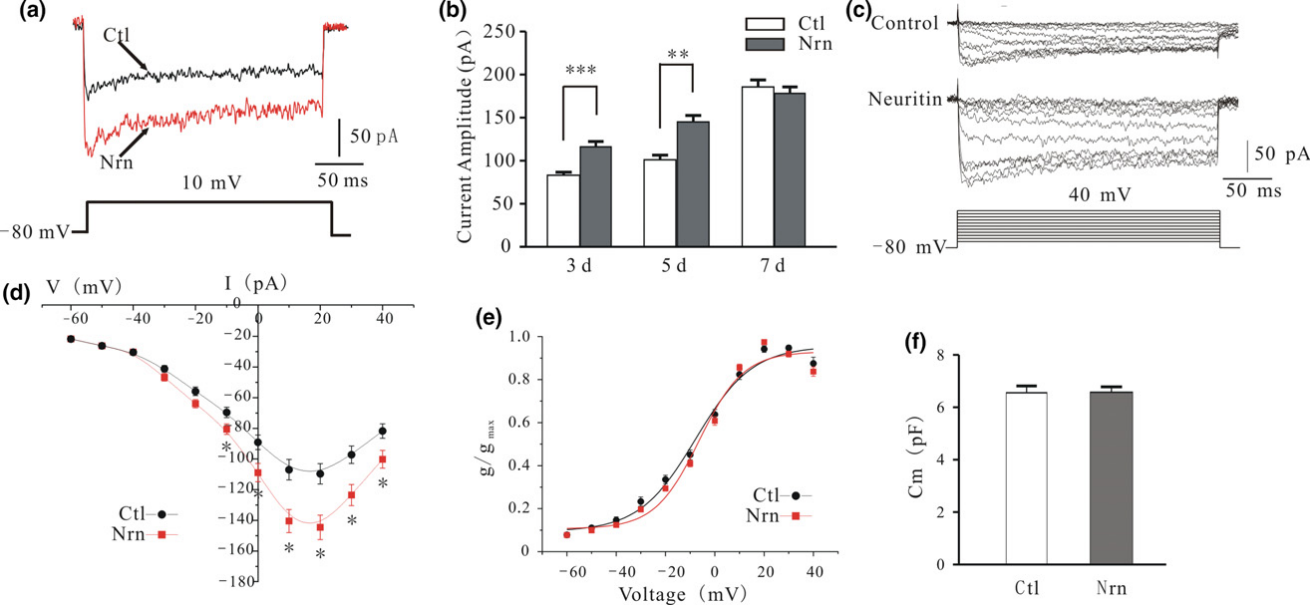
Neuritin increases *I*_Ca_ amplitude without altering voltage‐gating properties of Ca^2+^ channels. (a) Representative current traces obtained from control and neuritin‐treated cerebellar granule neurons (CGNs). *I*_Ca_ was elicited by depolarization from a holding potential of −80 mV to 10 mV for 250 ms. (b) Quantification of *I*_Ca_ amplitude recorded from control and neuritin‐treated CGNs cultured for different lengths of time (3 days Ctl, *n* = 48; 3 days Nrn, *n* = 31; 5 days Ctl, *n* = 48; 5 days Nrn, *n* = 37; 7 days Ctl, *n* = 36; 7 days Nrn, *n* = 21). (c) Representative traces obtained from control and neuritin‐treated CGNs with a steady‐state voltage protocol. (d) Voltage‐dependent activation curve of *I*_Ca_ in CGNs cultured in the absence or presence of neuritin (Ctl, *n* = 43; Nrn, *n* = 36). (e) Plot of normalized conductance as a function of command potential in CGNs cultured in the absence or presence of neuritin (Ctl, *n* = 21; Nrn, *n* = 28). Data points were fitted with a sigmoidal function using the Boltzmann model. (f) Quantitative analysis of the effects of neuritin on membrane capacitance (Ctl, *n* = 48; Nrn, *n* = 37). Data represent mean ± SEM, *n* indicates the number of cells. *p < 0.05, **p < 0.01, ***p < 0.001 between two groups connected with a straight line (unpaired t test or one‐way anova followed by Bonferroni post hoc test).

Ca^2+^ influx after depolarization occurs through VGCCs (Kirischuk *et al*. 1996; Toescu 1999). We recorded the VGCC current (*I*_Ca_) by patch‐clamping 5 DIC CGNs to determine whether VGCCs were activated by neuritin. Culture medium was replaced by recording bath solution with 150 ng/mL neuritin also for 20 min. *I*_Ca_ was elicited by depolarization from a holding potential of −80 mV to 10 mV for 250 ms (Fig. 5a). Neuritin increased *I*_Ca_ amplitude in CGNs in a time‐dependent manner (Fig. 5b) by 39.7 ± 5.7% (*n* = 48 and 31, *p* < 0.001) and 43.5 ± 6.3% (*n* = 48 and 37, *p* < 0.001) after 3 and 5 DIC, respectively, but not after 7 DIC (6.3 ± 4.2%, *n* = 36 and 21, *p* > 0.05). The current–voltage (*I*–*V*) curves obtained by delivering 250‐ms depolarizing pulses from a holding potential of −80 mV to between −60 and 40 mV in 10‐mV steps at 10‐s intervals (Fig. 5c) revealed that *I*_Ca_ was increased from a negative potential of −60 mV to a maximum value of 10 mV (Fig. 5d). We then evaluated steady‐state *I*_Ca_ activation by measuring membrane conductance of Ca^2+^ (*g*_Ca_) which was determined with the equation *g*_Ca_ =* I*_Ca_/(*V*_m_ − *V*_rev_), where *V*_m_ is the membrane potential and *V*_rev_ is the reversal potential for Ca^2+^. We then plotted *g*_Ca_/*g*_Ca‐max_ against membrane potential and fitted the line with a sigmoidal function using the Boltzmann model—that is, *g*_Ca/_ *g*_Ca‐max_ = 1/ {1 + exp [(*V*_m1/2_ − *V*_m_)/*k*]}. The half‐activation potential (the voltage at which the current amplitude is half‐activated) in the absence or presence of neuritin obtained from the *g*–*V* curve was −7.8 ± 1.0 mV (*n* = 21) and −5.8 ± 0.7 mV (*n* = 28), respectively (*p* > 0.05, Fig. 5e). We also compared membrane capacitance between neuritin treatment (6.9 ± 0.3 pF, *n* = 48) and control (6.6 ± 0.3 pF, *n* = 37) groups and found no significant difference (*p* > 0.05, Fig. 5f). Taken together, these results indicate that neuritin increases *I*_Ca_ amplitude without altering the voltage‐gating properties of *I*_Ca_ channels or membrane capacitance.

**Figure 6:**
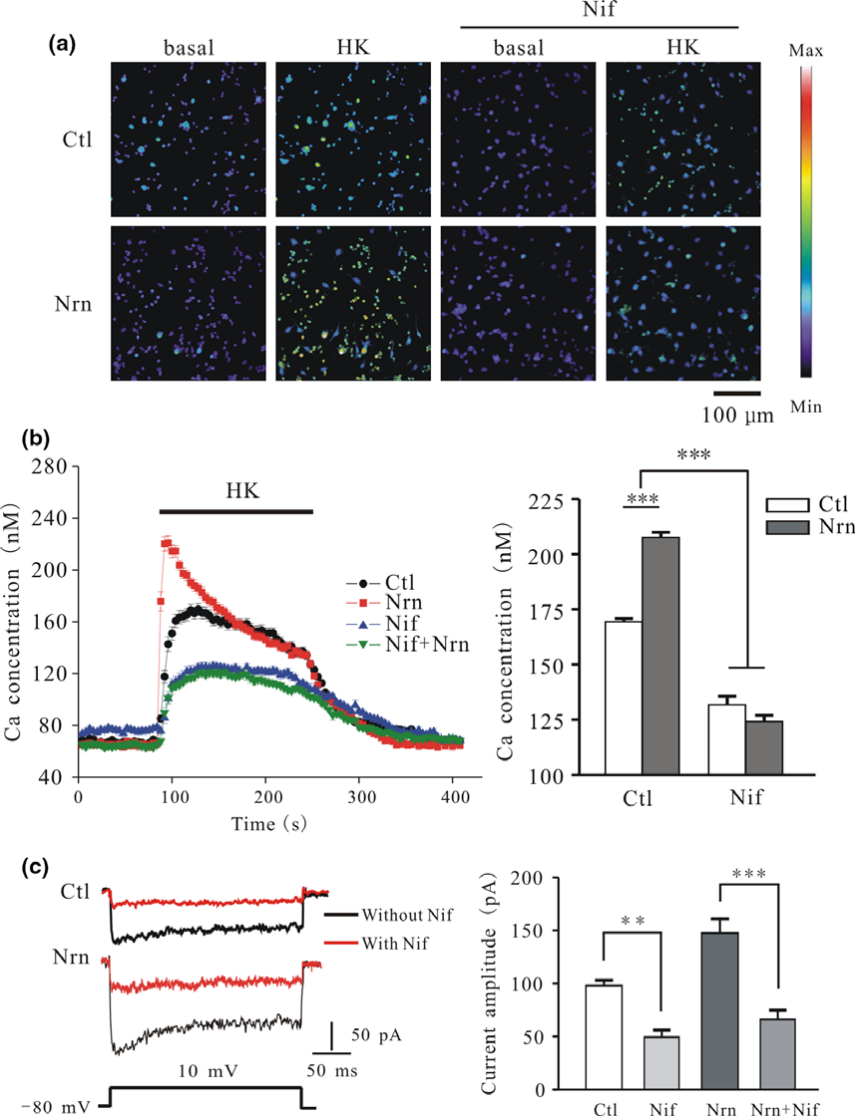
Blocking L‐type voltage‐gated calcium channels with nifedipine inhibits neuritin‐induced increase in [Ca^2+^]_i_ and voltage‐activated *I*_Ca_ amplitude in cerebellar granule neurons (CGNs). (a) Ca^2+^ imaging before and after depolarization by high potassium (K^+^) (HK) solution in neuritin‐treated CGNs cultured in the absence or presence of nifedipine (Nif). (b) Changes in [Ca^2+^]_i_ upon application of a depolarizing stimulus (left) and quantitative analysis of [Ca^2+^]_i_ (right) in CGNs cultured in the absence or presence of nifedipine with or without neuritin treatment (Ctl, *n* = 149; Nrn, *n* = 122; Nif, *n* = 58; Nif + Nrn, *n* = 60). (c) Representative current traces (left) and quantitative analysis (right) of the effect of nifedipine perfusion for 5 min on neuritin‐induced increase in *I*_Ca_ amplitude (Ctl, *n* = 8; Nrn, *n* = 8; Nif, *n* = 8; Nif + Nrn, *n* = 8). Data represent mean ± SEM, *n* indicates the number of cells. **p < 0.01, ***p < 0.001 between two groups connected with a straight line (one‐way anova followed by Bonferroni post hoc test).

**Figure 7:**
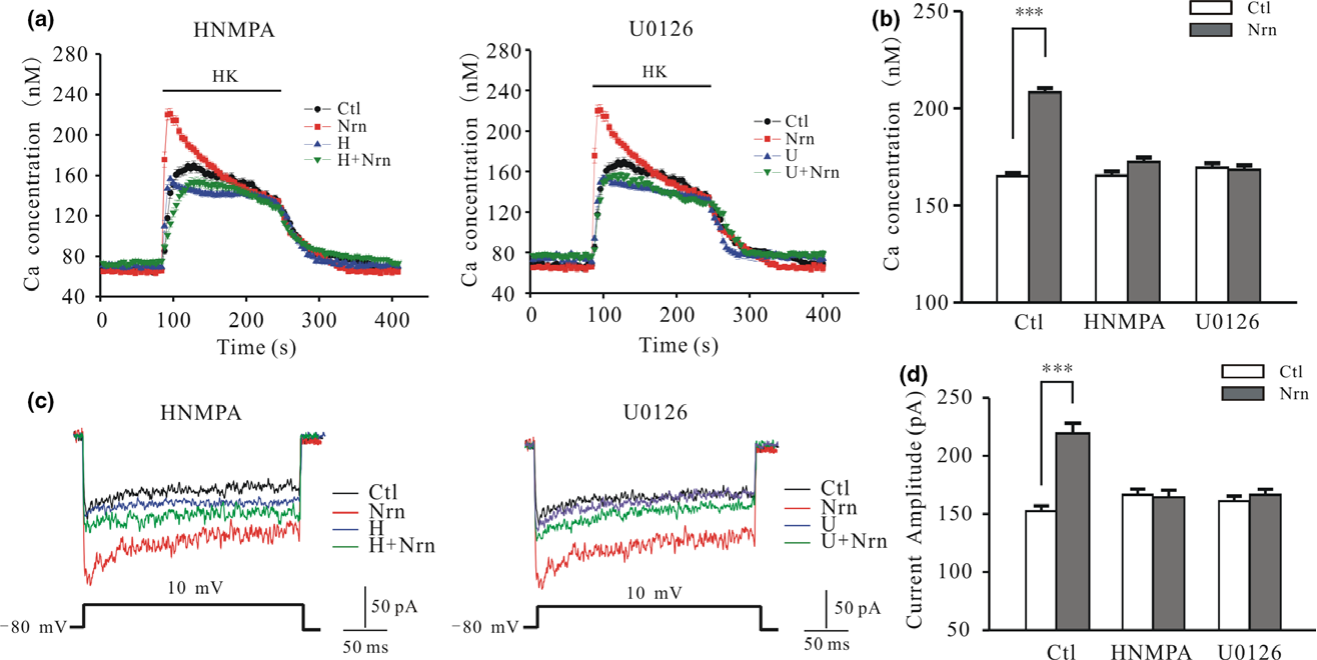
Blocking IR and extracellular signal‐regulated kinase activity inhibits neuritin‐induced increase in [Ca^2+^]_i_ and *I*_Ca_ amplitude in cerebellar granule neurons (CGNs). (a) Changes in [Ca^2+^]_i_ in CGNs upon application of a depolarizing stimulus in the absence or presence of hydroxy‐2‐naphthalenylmethyl phosphonic acid (HNMPA) (H) and U0126 (U) with or without neuritin treatment. (b) Quantitative analysis of the effects of HNMPA or U0126 on the neuritin‐induced increase in [Ca^2+^]_i_ (Ctl, *n* = 120; Nrn, *n* = 120; HNMPA, *n* = 94; HNMPA + Nrn, *n* = 86; U0126, *n* = 65; U0126 + Nrn, *n* = 91). (c) Representative current traces obtained from control and neuritin‐treated CGNs cultured in the absence or presence of HNMPA and U0126. (d) Quantitative analysis of the effects of neuritin on *I*_Ca_ amplitude in CGNs cultured in the absence or presence of HNMPA and U0126 (Ctl, *n* = 44; Nrn, *n* = 34; HNMPA, *n* = 25; HNMPA + Nrn, *n* = 34; U0126, *n* = 27; U0126 + Nrn, *n* = 31). Data represent mean ± SEM, *n* indicates the number of cells. ***p < 0.001 between two groups connected with a straight line (one‐way anova followed by Bonferroni post hoc test).

Our previous study showed that L‐type Ca^2+^ channels are the main VGCCs up‐regulated by neurotrophic factors (Lu *et al*. 2016). To determine whether L‐type Ca^2+^ channels are responsible for the increases in Ca^2+^ influx and *I*_Ca_ amplitude induced by neuritin, CGNs were treated with the selective blocker nifedipine. Pre‐incubation or perfusion of CGNs with nifedipine inhibited the neuritin‐induced increases in HK‐evoked [Ca^2+^]_i_ and *I*_Ca_ amplitude, respectively (Fig. 6a–c). In the presence of 10 μM nifedipine (Lu *et al*. 2016), the HK‐evoked [Ca^2+^]_i_ peak decreased to 131.8 ± 3.8 and 124.2 ± 2.8 nM (*n* = 58 and 60, *p* > 0.05) (Fig. 6a and b). Consistent with the results from Ca^2+^ imaging, nifedipine perfusion for 5 min reduced *I*_Ca_ amplitude of the control and neuritin groups by 49.6 ± 6.0% and 55.2 ± 7.6% (Fig. 6c), respectively, suggesting that L‐type Ca^2+^ channels mediate the neuritin‐induced increase in *I*_Ca_ amplitude and [Ca^2+^]_i_. Moreover, blocking IR and ERK with 100 μM HNMPA and 10 μM U0126, respectively, abrogated the increase in Ca^2+^ influx and *I*_Ca_ amplitude induced by neuritin (Fig. 7a–d).

**Figure 8:**
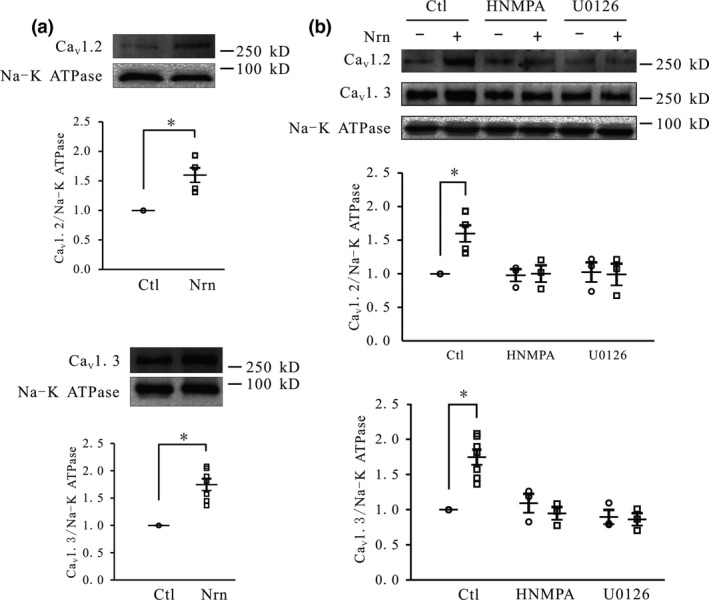
Neuritin enhances cell surface expression of Ca_V_1.2 and Ca_V_1.3 in cerebellar granule neurons (CGNs), which is inhibited by hydroxy‐2‐naphthalenylmethyl phosphonic acid (HNMPA) and U0126. (a) Western blot and quantitative analyses of the effects of neuritin on cell surface expression of Ca_V_1.2 (top) and Ca_V_1.3 (bottom) in CGNs (*n* = 6). (b) Western blot and quantitative analyses of the effects of HNMPA and U0126 on neuritin‐induced increases in Ca_V_1.2 and Ca_V_1.3 expression in CGNs (*n* = 3). Data represent mean ± SEM, *n* indicates the number of experiments. *p < 0.05 between two groups connected with a straight line (unpaired t test or one‐way anova followed by Bonferroni post hoc test).

We examined whether the neuritin‐mediated increase in *I*_Ca_ is because of an increase in channel expression at the cell membrane. Ca_V_1.2 and Ca_V_1.3 are the major α subunits of L‐type Ca^2+^ channels in the central nervous system (Forti and Pietrobon 1993; Koschak *et al*. 2007). We used biotin labeling to quantify the expression of Ca_V_1.2 and Ca_V_1.3 proteins on the membrane of CGNs following neuritin treatment. Ca_V_1.2 and Ca_V_1.3 levels were increased by 59.9 ± 12.4% (*n* = 6, *p* < 0.05) and 74.8 ± 11.8% (*n* = 6, *p* < 0.05), respectively, in the presence of neuritin, as determined by western blotting (Fig. 8a). Consistent with the observed effects of neuritin on HK depolarization‐induced Ca^2+^ influx and *I*_Ca_ amplitude, the cell membrane expression of Ca_V_1.2 and Ca_V_1.3 was blocked by HNMPA and U0126 (Fig. 8b).

### Ca^2+^/CaM/CaN/NFATc4 signaling mediates increases in neurite length and spine density induced by neuritin

**Figure 9:**
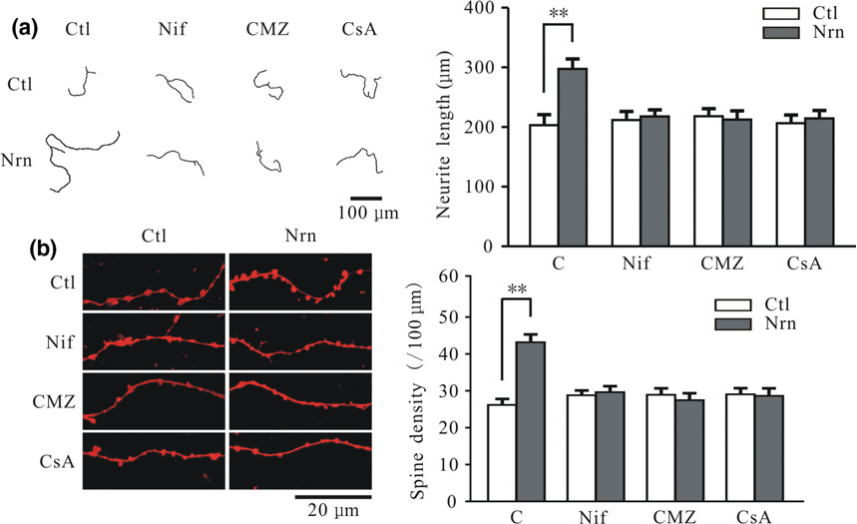
Nifedipine, calmidazolium chloride (CMZ), and cyclosporin A (CsA) treatments inhibit neuritin‐induced increases in cerebellar granule neuron (CGN) neurite length and spine density. (a) Representative images and quantitative analysis of the effects of neuritin on neurite length in CGNs cultured in the absence or presence of nifedipine, CMZ, and CsA (Ctl, *n* = 41; Nrn, *n* = 52; Nif, *n* = 34; Nif + Nrn, *n* = 39; CMZ, *n* = 38; CMZ + Nrn, *n* = 43; CsA, *n* = 39; CsA + Nrn, *n* = 46). (b) Representative images and quantitative analysis of the effects of neuritin on total spine density in CGNs cultured in the absence or presence of nifedipine, CMZ, and CsA (Ctl, *n* = 28; Nrn, *n* = 35; Nif, *n* = 29; Nif + Nrn, *n* = 23; CMZ, *n* = 20; CMZ + Nrn, *n* = 25; CsA, *n* = 23; CsA + Nrn, *n* = 20). Results represent mean ± SEM, *n* indicates the number of cells. **p < 0.01 between two groups connected with a straight line (one‐way anova followed by Bonferroni post hoc test).

Ca^2+^ and CaM play critical roles in regulating the growth and maturation of neurites and their spines (Borodinsky *et al*. 2002; Gaudilliere *et al*. 2004). We investigated whether Ca^2+^ and CaM are involved in the neuritin‐induced increases in neurite length and spine density. We found that these effects were abrogated by blocking L‐type channels with nifedipine and by inhibiting CaM and CaN by treatment with CMZ and CsA, respectively (Fig. 9a and b). Application of 10 μM nifedipine, 2 μM CMZ, or 5 μM CsA (Yao *et al*. 2016) could eliminate the neuritin‐induced increases in neurite length and spine density (Fig. 9a and b).

**Figure 10:**
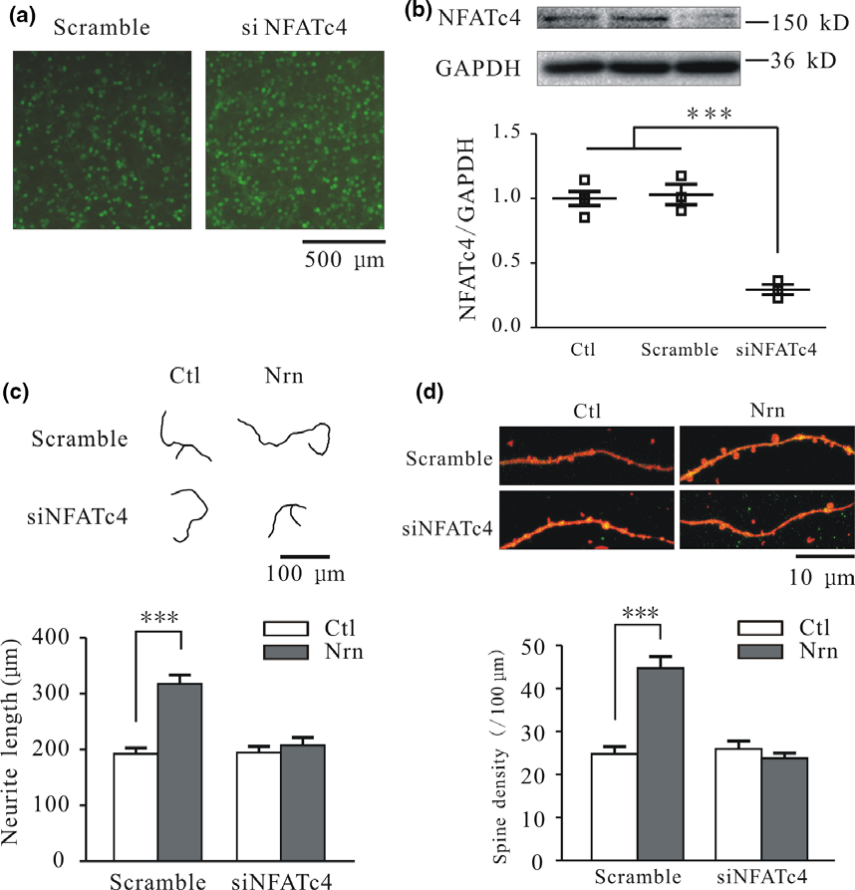
Nuclear factor of activated T cells (NFAT)c4 silencing abrogates neuritin‐induced increases in cerebellar granule neuron (CGN) neurite length and spine density. (a) Micrographs showing the transfection efficiency of lentivirus expressing green fluorescent protein and a scrambled control sequence (Scramble) or siNFATc4. (b) Western blot and quantitative analyses of the suppression of NFATc4 expression by siNFATc4 (*n* = 3). (c and d) Representative images and quantification of the effects of neuritin on neurite length (c) (Scramble, *n* = 20; Scramble + Nrn, *n* = 20; siNFATc4, *n* = 22; siNFATc4 + Nrn, *n* = 17) and total spine density (d) (Scramble, *n* = 22; Scramble + Nrn, *n* = 19; siNFATc4, *n* = 19; siNFATc4 + Nrn, *n* = 19) in CGNs cultured in the absence or presence of siNFATc4. Results represent mean ± SEM, *n* indicates the number of experiments in western blot or number of cells in morphological analysis. ***p < 0.001 between two groups connected with a straight line (one‐way anova followed by Bonferroni post hoc test).

NFATc family transcription factors (NFATc1–4) are downstream effectors in the Ca^2+^/CaN pathway (Sato *et al*. 2005; Vashishta *et al*. 2009). Our previous work showed that neuritin can activate this pathway and induce the expression of K^+^ channels, thereby increasing neuronal excitability (Yao *et al*. 2016). In this study, we used a lentivirus expressing siNFATc4 to silence NFATc4 expression and determine its role in the stimulation of neuritogenesis in CGNs. The high transfection efficiency of lentivirus expressing green fluorescent protein along with a scrambled sequence or siNFATc4 was confirmed by fluorescence microscopy (Fig. 10a). Treatment of CGNs with the siNFATc4 lentivirus for 3 days reduced NFATc4 expression by 71.4 ± 8.5% relative to cells infected with the lentivirus expressing the scrambled sequence (*p* < 0.001, *n* = 3) (Fig. 10b). siNFATc4 treatment alone did not affect neurite length, but suppressed the neuritin‐induced increase in neurite length, which did not differ between the siNFATc4 only and siNFATc4 plus neuritin groups (*n* = 22 and 17, *p* > 0.05) but differed between the scrambled sequence plus neuritin and the siNFATc4 plus neuritin groups (*n* = 20 and 17, *p* < 0.001) (Fig. 10c). The neuritin‐induced increase in total spine density was also suppressed by siNFATc4 treatment, with no difference observed between the siNFATc4 and siNFATc4 plus neuritin groups (*n* = 19 and 19, *p* > 0.05), although the scrambled sequence plus neuritin and siNFATc4 plus neuritin groups differed significantly (*n* = 19 and 19, *p* < 0.001) (Fig. 10d).

## Discussion

We previously reported that neuritin increased *I*_A_ in rat CGNs by increasing the expression of K_V_4.2 and enhanced glutamate release and miniature excitatory post‐synaptic current frequency in mouse cortical neurons via up‐regulation of the Ca_V_3.3 subunit of T‐type VGCCs at the membrane surface, with activation of IR and ERK signaling mediating these effects (Yao *et al*. 2012; Lu *et al*. 2017). In this study, we found that neuritin increased neurite length and spine density in CGNs via the same receptors and signaling pathways through up‐regulation of the Ca_V_1.2 and Ca_V_1.3 subunits of L‐type VGCCs at the membrane surface, which enhanced Ca^2+^/CaN/NFATc4 signaling.

Although previous studies have suggested that neuritin functions as a neurotrophic factor to stimulate neurite growth (Naeve *et al*. 1997; Marron *et al*. 2005; Cappelletti *et al*. 2007), there is little information on how it induces the formation of neurites and spines in CGNs. We confirmed that neuritin has this effect in CGNs, which is consistent with what is known of CGN development (Powell *et al*. 1997). We noted that neuritin exerted variable effects on spine density depending on spine morphology and primarily increased the number of mushroom spines, which are mature and mediate more robust synaptic transmission as the so‐called memory spines (Bourne and Harris 2007). Thus, neuritin not only increases spine density but may also promote spine maturation in CGNs. It has been proposed that thin and mushroom spines are interconvertible and that long‐lasting enhancement of synaptic transmission enlarges the head size of thin spines, transforming them into stable mushroom (Matsuzaki *et al*. 2004). Given our observation that neuritin increased the total number of spines without reducing thin spine density, it is likely that it acts not by stimulating the interconversion of thin and mushroom spines, but by promoting mushroom spine formation, leading to enhanced synaptic plasticity.

An elevation in [Ca^2+^]_i_ is critical for activation of Ca^2+^/CaM‐dependent neurite growth and synapse maturation in hippocampal neurons and CGNs (West *et al*. 2002). [Ca^2+^]_i_ is increased via Ca^2+^ influx through VGCCs that are opened by HK stimulation (Kirischuk *et al*. 1996; Toescu 1999) or by an increase in basal [Ca^2+^]_i_, which is known to depend on calcium released from intracellular stores (Velazquez‐Marrero *et al*. 2014). Our results indicate that neuritin only enhanced HK‐induced increases in [Ca^2+^]_i_ by up‐regulating the expression of L‐type VGCCs on the membrane without altering basal [Ca^2+^]_i_. This is in agreement with the observation that the development of neurites and synapses in CGNs is modulated by HK‐induced depolarization and Ca^2+^ signaling (Borodinsky *et al*. 2002; Sato *et al*. 2005), and is consistent with our previous study showing that neurotropic factors or hormones such as growth/differentiation factor‐15 and melatonin increased HK‐induced Ca^2+^ influx but not basal [Ca^2+^]_i_ in rat CGNs (Liu *et al*. 2016; Lu *et al*. 2016). However, it also contradicts our previous finding in mouse CGNs, in which neuritin not only enhanced HK‐induced increases in [Ca^2+^]_i_ but also caused a slight increase in basal [Ca^2+^]_i_ (Yao *et al*. 2016). Additional studies are needed to determine whether there are species‐specific differences in the mechanisms of calcium release from intracellular stores in CGNs.

Various types of VGCC are expressed in immature and mature CGNs (Koschak *et al*. 2007). L‐type VGCCs are responsible for CaN‐mediated synaptogenesis (Sato *et al*. 2005), whereas N‐type channels are critical for CGN migration (Dangelo *et al*. 1994). The results presented here demonstrate that [Ca^2+^]_i_ following membrane depolarization and Nif‐sensitive *I*_Ca_ were increased by neuritin, suggesting the activation of L‐type VGCCs, although we cannot exclude the possibility that nifedipine‐insensitive Ca^2+^ channels or N‐, P/Q‐, R‐type Ca^2+^ channels are also involved. We found that *I*_Ca_ amplitude and HK‐evoked [Ca^2+^]_i_ in CGNs increased with developmental stage, which is consistent with observations in cerebellar slice cultures (Dangelo *et al*. 1994). That is, the neuritin‐induced increase in *I*_Ca_ amplitude and HK depolarization and resultant Ca^2+^ influx only occurred in CGNs after 3 and 5 DIC. This may be explained by the fact that CGN Ca^2+^ channels are more sensitive to neuritin at early developmental stages, and is consistent with the function of neuritin in promoting spine development and maturation in CGNs.

L‐type Ca channels include Ca_V_1.1–1.4 α subunits (Koschak *et al*. 2007). Ca_V_1.1 and 1.4 are mainly expressed in skeletal muscle and retinal cells, whereas Ca_V_1.2 and Ca_V_1.3 are abundant in the brain where they regulate neuronal excitability, synaptic plasticity, and activity‐dependent gene transcription (Forti and Pietrobon 1993; Koschak *et al*. 2007). Although Ca_V_1.2 and Ca_V_1.3 α subunits differ in their biophysical properties, brain distribution, and function (Tuckwell 2012; Berger and Bartsch 2014), our western blot analysis showed that Ca_V_1.2 and Ca_V_1.3 α subunits were similarly up‐regulated by neuritin, which is consistent with a previous finding that functionally distinct L‐type Ca^2+^ channels coexist in rat CGNs (Forti and Pietrobon 1993). Increased surface expression of Ca_V_1.2 and Ca_V_1.3 α subunits can also explain why neuritin treatment increased *I*_Ca_ amplitude but did not affect L‐type VGCC gating properties.

**Figure 11:**
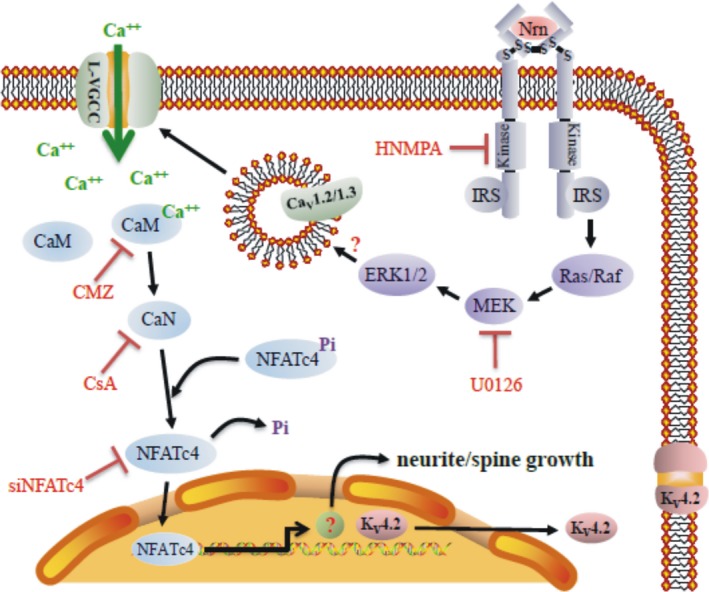
Schematic illustration depicting the mechanisms of neuritin in the up‐regulation of the Ca_V_1.2 and Ca_V_1.3 subunits of L‐type voltage‐gated calcium channels (VGCCs) on the cell membrane, and the subsequent increase in neurite length, spine density, and K_V_4.2 expression. IRS, insulin receptor substrate.

Similar to our earlier studies examining the effect of neuritin on K_V_4.2 expression in rat CGNs and T‐type Ca_V_3.3 membrane expression in mouse cortical neurons (Yao *et al*. 2012; Lu *et al*. 2017), neuritin‐induced effects on spine density, *I*_Ca_ amplitude, and Ca^2+^ influx elicited by HK were mimicked by insulin and abolished by inhibition of IR and ERK signaling, suggesting that these pathways mediate the activities of neuritin. We also found that neuritin‐induced increases in Ca_V_1.2 and Ca_V_1.3 expression were dependent on IR and ERK signaling. ERK can directly phosphorylate ion channel subunits and may alter the gating properties of K^+^ channels in the same manner that neurotransmitter regulates *I*_A_ (Hu *et al*. 2007). Since neuritin did not alter the gating properties of Ca^2+^ channels but instead increased Ca_V_1.2 and Ca_V_1.3 expression on the cell membrane, we propose that activation of ERK signaling by neuritin enhances Ca_V_1.2 and Ca_V_1.3 trafficking to the membrane in the same way that it increases cell surface T‐type VGCC Ca_V_3.3 expression via activation of ERK signaling in mouse cortical neurons (Lu *et al*. 2017). It is likely that neuritin‐induced activation of IR and ERK signaling caused the up‐regulation of the Ca_V_1.2 and Ca_V_1.3 subunits of L‐type VGCCs on the cell membrane, increased Ca^2+^ influx, and activated the Ca^2+^/CaN/NFATc4 pathway (Fig 11).

NFATc1–4 are key downstream effectors of CaN signaling (Sato *et al*. 2005; Vashishta *et al*. 2009). NFATc4 plays an important role in neural development, axon elongation, and neuronal survival (Benedito *et al*. 2005; Sato *et al*. 2005). We previously showed that neuritin along with Ca^2+^ and CaN activated NFATc4 to increase K_V_4.2 promoter activity in CGNs, which enhanced *I*_A_ and neuronal excitability (Yao *et al*. 2016). Here we found that blocking *I*_Ca_ channels, abolishing CaM or CaN function, or silencing NFATc4 abolished the effects of neuritin on neurite length and spine density, suggesting that these are mediated by Ca^2+^, CaN, and NFATc4. However, since neurite growth and spine maturation induced by Ca^2+^ signaling involve a variety of mechanisms such as regulation of cargo loading and stabilization of the cytoskeleton and post‐synaptic densities (Ichinose *et al*. 2015; Ji *et al*. 2017; Yadav *et al*. 2017), additional studies are required to identify the genes related to neurite and spine growth that are up‐regulated by neuritin/Ca^2+^/CaN /NFATc4.

*I*_A_ is the predominant K^+^ current in many mature neurons (Kanold and Manis 1999; Tkatch *et al*. 2000; Plant *et al*. 2006) that is initially activated at the sub‐threshold range of membrane potential and was previously implicated in the control of neuronal excitability (Carrasquillo *et al*. 2012; Yang *et al*. 2014). Interestingly, the expression of K_V_4.2 increased with the duration of the micro‐explant and CGN culture period (Shibata *et al*. 1999; Yao *et al*. 2012), and *I*_A_ was developmentally regulated and associated with the maturation and migration of CGNs (Shibata *et al*. 2000; Yao *et al*. 2013). Notably, neuritin induced increases in *I*_A_ and K_V_4.2 expression from day 3–5 of culture (Yao *et al*. 2012), which corresponded to the neuritin‐induced increase in neurite and spine growth. We speculate that the increased expression of K_V_4.2 induced by neuritin may not be directly related to its effect on spine growth. Alternately, increased expression of K_V_4.2 and spine growth may both require neuritin‐induced increases in calcium influx and Ca^2+^/CaN/NFATc4 pathway activation, which may occur simultaneously during enhancement of CGN development and maturation induced by neuritin (Fig. 11).

In conclusion, we demonstrated that neuritin promotes neurite and spine growth via Ca^2+^‐mediated activation of CaN and NFATc4. However, while depolarization did not affect the development of immature dendrites, it was shown to prevent terminal maturation of CGNs through activation of CaN in cultured cerebellar slices (Okazawa *et al*. 2009). Moreover, dendrite maturation was blocked by depolarization and NFATc4 activation in cultured CGNs (Ding *et al*. 2013). It is possible that the inconsistency between these results and ours is because of the fact that we used 25 mM rather than 5 mM K^+^ medium for the cultured rat CGNs. Further study is needed to clarify the role of K^+^ concentration in the neuritin‐induced effects on neurite and spine development in cultured CGNs.

## Open science badges

This article has received a badge for *Open Materials* because it provided all relevant information to reproduce the study in the manuscript. The complete Open Science Disclosure form for this article can be found at the end of the article. More information about the Open Practices badges can be found at https://cos.io/our-services/open-science-badges/.
